# Repeatability and Reproducibility of Pancreas Volume Measurements Using MRI

**DOI:** 10.1038/s41598-020-61759-9

**Published:** 2020-03-16

**Authors:** Jonathan M. Williams, Melissa A. Hilmes, Branch Archer, Aidan Dulaney, Liping Du, Hakmook Kang, William E. Russell, Alvin C. Powers, Daniel J. Moore, John Virostko

**Affiliations:** 1 Department of Medicine, Division of Diabetes, Endocrinology, and Metabolism, Vanderbilt University Medical Center, Nashville, Tennessee USA; 2 Department of Radiology and Radiological Sciences, Vanderbilt University Medical Center, Nashville, Tennessee USA; 3 Department of Pediatrics, Vanderbilt University Medical Center, Nashville, Tennessee USA; 4 Department of Electrical and Computer Engineering, University of Texas at Austin, Austin, Texas USA; 5 Department of Diagnostic Medicine, Dell Medical School, University of Texas at Austin, Austin, Texas USA; 6 Department of Biostatistics, Vanderbilt University Medical Center, Nashville, Tennessee USA; 7 Department of Cell and Developmental Biology, Vanderbilt University, Nashville, Tennessee USA; 8 Department of Molecular Physiology and Biophysics, Vanderbilt University, Nashville, Tennessee USA; 9 VA Tennessee Valley Healthcare System, Nashville, Tennessee USA; 10 Department of Pathology, Immunology, and Microbiology, Vanderbilt University, Nashville, Tennessee USA; 11 Livestrong Cancer Institutes, Dell Medical School, University of Texas at Austin, Austin, Texas USA; 12 Department of Oncology, Dell Medical School, University of Texas at Austin, Austin, Texas USA

**Keywords:** Imaging techniques, Type 1 diabetes

## Abstract

Reduced pancreas volume, as measured by non-contrast magnetic resonance imaging (MRI), is observed in individuals with newly-diagnosed type 1 diabetes (T1D) and declines over the first year after diagnosis. In this study, we determined the repeatability and inter-reader reproducibility of pancreas volume measurements by MRI. Test-retest scans in individuals with or without T1D (n = 16) had an intraclass correlation coefficient (ICC) of 0.985 (95% CI 0.961 to 0.995) for pancreas volume. Independent pancreas outlines by two board-certified radiologists (n = 30) yielded an ICC of 0.945 (95% CI 0.889 to 0.973). The mean Dice coefficient, a measurement of the degree of overlap between pancreas regions of interest between the two readers, was 0.77. Prandial state did not influence pancreatic measurements, as stomach volume did not correlate with pancreas volume. These data demonstrate that MRI measurements of pancreas volume between two readers are repeatable and reproducible with ICCs that correspond to excellent clinical significance (ICC > 0.9), are not related to changes in stomach volume, and could be a useful tool for clinical investigation of diabetes and other pancreas pathologies.

## Introduction

Pancreas volume is lower in individuals with type 1 diabetes (T1D)^1–4^ and may also be reduced in type 2 diabetes^5^. Reduced pancreas volume, as measured by non-contrast magnetic resonance imaging (MRI), is found in newly-diagnosed T1D^6–8^ and declines over the first year after diagnosis^7^. Reduction in pancreas volume is also observed in individuals without overt diabetes who are positive for 2 or more T1D-related autoantibodies and have a higher risk for developing T1D^7,8^. These recent findings raise the possibility that a reduction in pancreas volume may be useful in studying T1D and challenges the paradigm that T1D is solely an islet cell disorder. However, the repeatability and reproducibility of MRI measurements of the pancreas are not well established. Thus, it is currently difficult to determine whether longitudinal changes in pancreas volume reflect true changes in pancreas size or reflect measurement variability. For MRI to be utilized to accurately measure pancreas volume and track longitudinal changes in individuals, the variation between measurements of pancreas volume and factors affecting those measurements must be quantified.

Previous studies on the repeatability of multiple pancreas volume measurements in the same individual were limited by small sample sizes^9,10^. Similarly, studies of inter-reader reliability, or how well readers agree when measuring the same pancreas, were likewise performed in small cohorts^8^. Furthermore, prior studies did not examine the repeatability and reproducibility of pancreas volume measurements in individuals with diabetes and controls to assess the influence of diabetes on these metrics. We sought to quantify both the intra- and inter-reader repeatability in larger cohorts using images acquired with the same parameters to facilitate comparison of intra- and inter-reader agreement in individuals with and without diabetes.

The relationship between stomach volume, prandial state, and the effect on pancreas volume is not well defined and could contribute to variability in measurements of pancreas volume. The stomach, a distensible organ, can expand upon feeding from its empty state of approximately 0.08 L to up to 4 L^11^. This large expansion of the stomach upon feeding could compress or displace surrounding organs such as the pancreas. One study in canines found that fasting did not confound use of abdominal ultrasound^12^, while others have reported that food in the gastrointestinal tract impairs pancreas visibility^13^. Some imaging studies of human pancreas have fasted participants prior to imaging^6,8^ while others have not^7,14^. Thus, the influence of oral intake and stomach volume on pancreas volume is not well-characterized and might lead to discrepancies between studies performed in the fed and fasted state. Furthermore, requiring fasting prior to MRI complicates imaging protocols and may be especially problematic in individuals with diabetes.

This study sought to determine the repeatability of pancreas volume measurements to establish a threshold for differentiating true changes in pancreas volume from variability in measurements. Repeatability was assessed by same-day test-retest measurements in the same individual. Inter-reader reliability of pancreas volume measurements was determined by independent outline of the same pancreas MR image by two board-certified radiologists. The relationship between stomach volume and pancreas volume was assessed in participants whose stomach and pancreas were both in the field of view, including multiple scans performed in individuals with variable stomach volume. We demonstrate that measurements of pancreas volume by MRI are repeatable and reproducible between two readers and are not influenced by stomach volume. These findings suggest that pancreas volume measurements by MRI could be useful for clinical investigation in diabetes and other pancreas pathologies.

## Results

### Measurements of pancreas volume are repeatable

**Figure 1:**
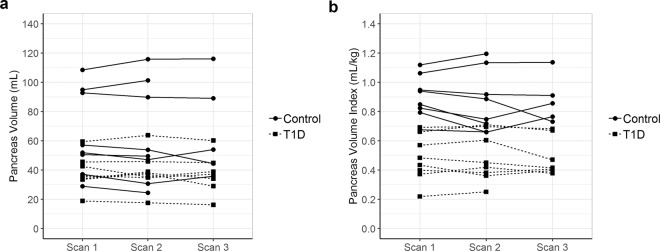
Measurements of pancreas volume are repeatable. Two or three repeat MRIs were performed on the same day in individuals with and without T1D (dashed and solid lines, respectively). Repeat measurements of pancreas volume (**a**) and pancreas volume index (**b**) are shown for each subject. Pancreas volume index was calculated by dividing the pancreas volume by the subject’s weight. Intraclass correlation coefficients (ICC) were reported with a 95% confidence interval.

To determine the test-retest repeatability of pancreas volume measurements, we performed two or three repeat MRIs on the same day in individuals with or without type 1 diabetes (n = 16, total) (Fig. 1). Study participant attributes were: 56% female, average age of 23.3 y, range 9–40 y, and average BMI of 25.2 kg/m^2^. The test-retest scans had an intraclass correlation coefficient (ICC) of 0.985 (95% CI 0.961–0.995) for pancreas volume (Fig. 1a) and an ICC of 0.955 (95% CI 0.889–0.985) for pancreas volume index (Fig. 1b), a metric which normalizes the pancreas volume for the individual’s weight.

The overall mean of the absolute difference among repeats within a subject was 3.8 mL (95% CI 2.9–4.8 mL by bootstrapping) while the overall median of the absolute difference among repeats within a subject was 4.3 mL (95% CI 2.3–4.9 mL by bootstrapping). The repeatability coefficient, which defines the magnitude of the maximum difference between repeated observations expected in 95% of paired observations, was 9.2 mL. A repeated measures ANOVA test did not find a statistically significant difference within repeat scans of the same individual (P = 0.59). Wilcoxon rank sum test compared differences in pancreas volume measurement between repeated scans for controls and T1Ds and found no significant difference (P = 0.3), suggesting that repeatability of pancreas volume measurement by MRI does not differ between individuals with or without T1D. These findings indicate that same day repeat MRI measurements of pancreas volume by a single radiologist in individuals with or without T1D are highly repeatable.

### Measurements of pancreas volume between two readers are reproducible

**Figure 2:**
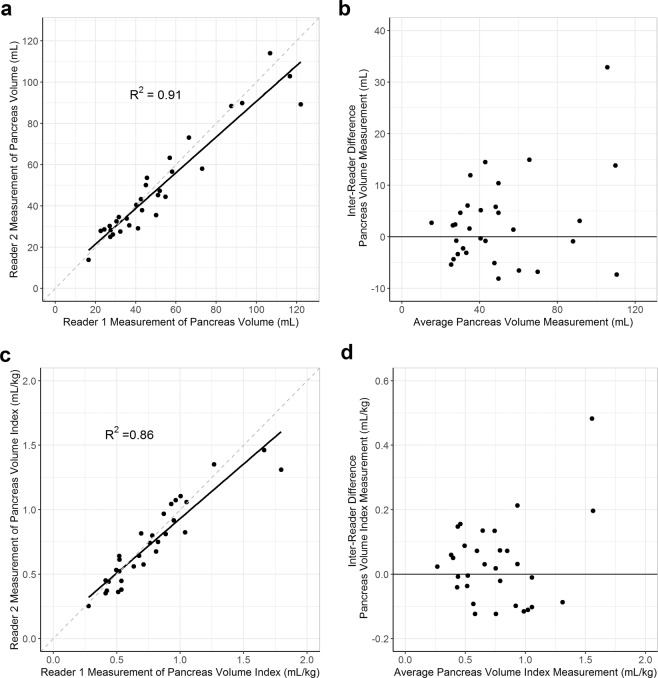
Measurements of pancreas volume between two readers are reproducible. Two board-certified radiologists independently outlined the pancreas of 30 different individuals (31 total scans). Correlation between measurements of pancreas volume (**a**) and pancreas volume index (**c**) between the two readers is shown. Bland–Altman plots displaying the differences in measurements between readers plotted against the average measurement taken for each subject are shown for both pancreas volume (**b**) and pancreas volume index (**d**). Pancreas volume index was calculated by dividing the pancreas volume by the subject’s weight. ICCs were reported with a 95% confidence interval.

Inter-reader reliability of pancreas volume measurements was determined by independent pancreas outline of the same MR image by two board-certified radiologists who were blinded to each other’s measurement and to the diabetes status of each participant (n = 30). Study participant attributes were: 47% female, average age of 21.1 y, and average BMI of 23.6 kg/m^2^. Comparing results from two readers yielded an ICC of 0.945 (95% CI 0.889–0.973) for pancreas volume (Fig. 2a) and an ICC of 0.923 (95% CI 0.848–0.962) for pancreas volume index (Fig. 2c), with no significant difference between pancreas size and reader agreement (P = 0.27) (Fig. 2b,d). Wilcoxon rank sum test compared differences in pancreas volume measurement between two readers for control and T1D participants and found no significant difference (P = 0.8).

**Figure 3:**
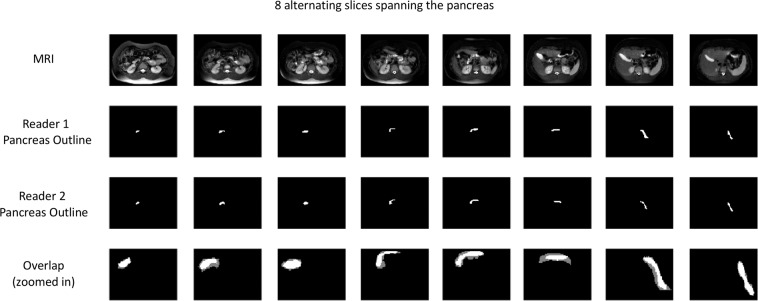
Outlines of pancreas by two readers of the same images overlap. The pancreas from a representative subject (Dice coefficient = 0.77) was outlined by two board-certified radiologists across eight alternating MRI slices of the pancreas. The overlap between the two readers’ pancreas outlines is shown in the bottom row and zoomed in for clarity. Regions of agreement are shown in bright white, while gray regions denote regions of non-overlap. The average Dice coefficient was 0.77 for the 31 scans analyzed.

The overall mean of the absolute difference between readers for a scan was 6.2 mL (95% CI 4.3–8.7 mL by bootstrapping), and the overall median of the absolute difference between readers for a scan was 4.7 mL (95% CI 3.1–6.1 mL by bootstrapping). A representative comparison of the two radiologists’ outlines is shown across eight alternating slices of the pancreas (Fig. 3). The mean Dice coefficient, a measure of the overlap between pancreatic regions of interest between the two radiologists, was 0.77. These results indicate that MRI measurements of pancreas volume between two different readers are reproducible, and this reproducibility is not impacted by T1D status.

### Stomach volume does not affect measurements of pancreas volume

**Figure 4:**
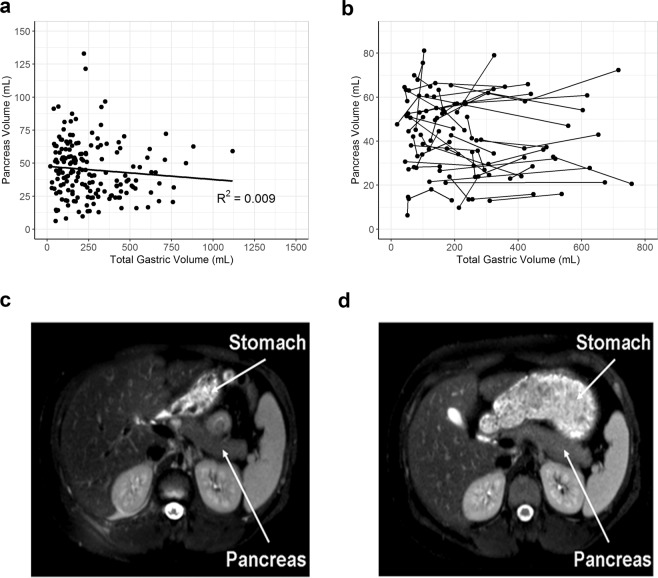
Stomach volume did not affect measurements of pancreas volume. (**a**) Correlation between measurements of pancreas volume and total stomach volume in individuals whose MRI data sets contained the entire stomach volume within the field of view (R^2^ = 0.009). (**b**) Individuals who had longitudinal measurements of pancreas and stomach volume. Representation of an individual with low stomach volume (**c**) and in an apparent fed state (**d**).

Stomach volume varied widely across study participants ranging from a low of 18 mL to a high of 1118 mL, reflecting a range of fed and fasted states. There was no correlation between stomach volume and pancreas volume across 173 scans (Fig. 4a) (R^2^ = 0.009). Participants who received multiple longitudinal MRIs on which the entire stomach volume was captured in the field of view (n = 45) displayed large changes in stomach volume (Fig. 4b). However, there was no correlation between these large changes in stomach volume and changes in pancreas volume. For instance, one participant received two MRI scans separated by six months on which the stomach volume ranged from 188 mL (Fig. 4c) to 716 mL (Fig. 4d), but pancreas volume was similar at each scan (65 mL vs 72 mL). This analysis indicates that stomach volume or prior oral intake does not influence MRI measurement of pancreas volume.

## Discussion

In this study we determined the repeatability of pancreas volume measurements using MRI. Study participants were either newly enrolled or retrospectively analyzed in our ongoing longitudinal MRI study, clinicaltrials.gov identifier NCT03585153^7^. We found that pancreas volume measurements were repeatable and reproducible with intraclass correlation coefficients in the “excellent” range^15^. Furthermore, we found no relationship between stomach volume and pancreas volume, indicating that stomach volume or prior oral intake does not affect MRI measurement of pancreas volume. Repeatability did not differ between individuals with T1D and normal controls. This validation represents a crucial step in operationalizing MRI for pancreas volume measurement and accurately tracking changes in volume over time, such as those demonstrated in early T1D^7^.

One intriguing alternative to the use of multiple readers is the use of machine learning techniques for computer-driven pancreas segmentation^16,17^. Automatic segmentation circumvents inter-reader variability, although we have found this variation to be modest. Presently, the accuracy of automated pancreas segmentation has lagged behind other abdominal organs^18^, likely due to the pancreas being a small, flexible abdominal organ with a high degree of variation among individuals in both shape and volume. A previous study found that Dice coefficients between computer models of pancreas segmentation and manual segmentation by radiologist were agreeable across the head, body, and tail of the pancreas^19^. Another study demonstrated reproducibility in a hybrid gradient, region growth and shape constraint segmentation method across multiple subjects^20^. Pancreas segmentation using machine learning is an area of active research and will be useful for screening large imaging datasets. This current study aids this development by establishing a benchmark for inter-reader agreement for comparison with automatic segmentation algorithms.

We found that stomach volume did not influence pancreas volume, suggesting that fasting or feeding does not affect measurement of pancreas volume using MRI. As fasting may complicate imaging in individuals with T1D who are prone to hypoglycemia, obviating the need for fasting will ease protocol compliance. Our findings agree with others who have shown that gastric conditions do not result in significant displacement of abdominal organs after feeding^21,22^. This study did not perform prospective fasting and feeding prior to MRI to determine whether pancreas volume measurements are influenced by peristaltic motion of the digestive tract or changes in duodenal inner lumen volume accompanying feeding.

A caveat of this study is that all images were obtained at one institution on a single MRI scanner. Efforts are underway to validate pancreas volume measurement across multiple sites using images acquired on different MRI platforms. The authors of this manuscript are collaborating to develop such a standard operating procedure, in the Multicenter Assessment of the Pancreas in Type 1 Diabetes (MAP-T1D) study, funded by the JDRF (NCT03585153). Standard operating procedures for pancreas imaging and reading will be developed as part of the MAP-T1D study and should aid in quantifying the reproducibility of MRI measurements acquired across MRI platforms. This analysis will accelerate multi-center application of pancreas volume measurements by MRI in future studies of pancreas pathology.

In summary, MRI measurement of pancreas volume is repeatable in scans performed on the same day in the same individual, reproducible between two readers of the same scan, and not affected by changes in stomach volume. Several studies have demonstrated that pancreas volume also changes in type 2 diabetes and chronic pancreatitis^23–26^, suggesting that pancreas volume, and relative changes in volume, may be an important biomarker across a range of pancreas pathologies. Changes in pancreas volume may provide a useful metric for observational and interventional trials in T1D.

## Materials and Methods

### Study participants

Study participants were either newly enrolled or retrospectively analyzed in our longitudinal MRI study (clinicaltrials.gov identifier NCT03585153). Some of the scans in this report were included in a publication that defined changes in pancreas volume in newly diagnosed (average duration = 2.2 months) type 1 diabetes (T1D)^7^. Individuals with T1D were recruited from a monthly class at the Eskind Diabetes Center at Vanderbilt University Medical Center, Nashville, TN. The clinical diagnosis of T1D was established by the participants’ endocrinologists based on the American Diabetes Association criteria for T1D. Healthy controls were recruited by flyers, word of mouth, or clinicaltrials.gov (NCT03585153) and screened to exclude diabetes or any other pancreas pathology. Additional exclusion criteria included presence of metallic implants contraindicated for MRI, current pregnancy or breastfeeding, or claustrophobia induced by the MRI bore. These studies were approved by the Vanderbilt University Institutional Review Board and performed in accordance with the guidelines and regulations set forth by the Human Research Protections Program. Informed consent was obtained from all participants and/or their legal guardians prior to MRI.

### MRI

MRI was performed on a Philips 3T Achieva scanner (Philips Healthcare, Best, The Netherlands) using a 16-channel receive torso coil. The image acquisition consisted of a T2-weighted fast-spin echo sequence with 1.5 × 1.5 × 5 mm spatial resolution and TR/TE/FA of 840 ms/70 ms/90°. Imaging was performed in the axial plane over two breath holds with a total imaging time of 25 s. SPAIR fat suppression was performed.

### Repeatability of pancreas volume measurements

To determine the test-retest repeatability of pancreas volume measurements by MRI, we performed two or three same-day repeat MRIs in the same individuals with or without T1D (n = 16). Briefly, participants were scanned the first time using a protocol including the T2-weighted fast-spin echo sequence. The participant then exited the scanner, stood and stretched, and returned into the scanner. A new survey was obtained, and then the T2-weighted fast-spin echo sequence MRI scan was repeated. For twelve participants, this process was immediately repeated one additional time, for a total of three MRIs.

All repeat scans were performed on the same day. The pancreas was outlined on axial slices by an experienced radiologist (M.A.H.) blinded to identifying information and the diabetes status of each participant. Regions of interest delineating the pancreas were created using MIPAV (NIH; https://mipav.cit.nih.gov). Axial areas within the region of interest were multiplied by the slice thickness (5 mm) and summed to yield total pancreas volume^10^. Pancreas volume index was calculated by dividing the pancreas volume by the subject’s weight^6,7^.

### Inter-reader reproducibility of pancreas volume measurements

Inter-reader reliability of pancreas volume measurements was determined through independent outline of the same pancreas MR images by two board-certified radiologists (M.A.H. and B.A.) who were blinded to each other’s measurement and to the diabetes status of each participant. M.A.H. has 14 years of experience as an attending radiologist in an academic setting and B.A. has 25 years of experience in private practice radiology. Scans were analyzed from 30 different individuals (31 total scans, as one person had two different scans included).

### Stomach volume

Participants were not given instructions about fasting prior to the MRI, and thus pancreas volume measurements included MRIs with variable stomach volumes. MRI data sets from our published cohort^7^ that contained the entire stomach volume within the field of view were retrospectively identified (n = 173). The MRI scans analyzed in this study of stomach volume included additional scans besides those analyzed in the intra- and inter-reader studies. The pancreas was outlined on axial slices by an experienced radiologist (M.A.H.) blinded to identifying information. The stomach was outlined on axial slices by an observer blinded to identifying information (A.D.). Stomach volume was calculated by multiplying the area of the bounded region by the thickness of each slice (5 mm) and summing the individual slices together.

### Statistical analysis

Statistical analysis was performed using R Statistical Software version 3.3.2 (R Foundation for Statistical Computing, Vienna, Austria). These analyses include intraclass correlation coefficients (ICC) that describe how strongly units of measurement in the same group resemble each other^15^. Additionally, the mean absolute difference within a subject’s repeated scans and the difference between two readers for a scan were calculated. The mean and median for all scans were reported with 95% confidence intervals by bootstrapping, a technique that estimates the variance or confidence interval of a statistical parameter through resampling of the same measurements without any distributional assumption. The root of the mean of squared difference across all study subjects (rMSD) was used to calculate the repeatability coefficient (r = 2.13 rMSD) for 16 subjects, which defines the magnitude of the maximum difference (absolute value) between repeated observations expected in 95% of paired observations (e.g. expected variability in pancreas volume measurement for a subject)^27^.

Bland-Altman plots were used to quantify agreement between the two different readers’ measurements of pancreas volume^28–30^. Spatial agreement between the two readers’ pancreas outlines was determined by the Dice coefficient, a method for defining the overlap between two unique regions of interest^31^. Repeated measures ANOVA was used to test for significant difference between repeated measurements of pancreas volume. Wilcoxon rank sum tests compared differences in pancreas volume measurement between two readers and between test-retest scans in individuals with and without T1D.

## Data Availability

The datasets generated during and/or analyzed during the current study are available from the corresponding author on reasonable request.
